# A Scoping Review of Clinical Features and Mechanisms of Orofacial Pain and Headache in Patients with Head and Neck Cancer

**DOI:** 10.3390/jcm14165722

**Published:** 2025-08-13

**Authors:** Ernesto Anarte-Lazo, Carlos Bernal-Utrera

**Affiliations:** 1 Faculty of Health, UNIE University, 28015 Madrid, Spain; ernesto.anarte@universidadunie.com; 2 Physiotherapy Department, Faculty of Nursing, Physiotherapy and Podiatry, University of Seville, 41009 Seville, Spain

**Keywords:** orofacial pain, headache, neoplasms, head and neck, pain measurement

## Abstract

Background: Orofacial pain (OFP) and headache are common and disabling conditions in people with head and neck cancer (HNC), although their clinical characteristics and underlying pain mechanisms remain poorly studied, leading to worse diagnosis and, thus, management. Therefore, this review aims to synthesize the literature regarding clinical features, pain descriptors, mechanisms, and assessment tools of OFP and/or headache in adults with HNC. Methods: A scoping review was conducted following the Arksey and O’Malley framework and reported using PRISMA-ScR guidelines. We searched PubMed, Embase, Scopus, and Web of Science. Quantitative and qualitative original studies were included. Data were charted and summarized using narrative synthesis. Results: Of 3647 records initially retrieved, 32 studies met the inclusion criteria. Most studies were observational and heterogeneous in design, population, and pain assessment methods. OFP was highly prevalent, with neuropathic descriptors (e.g., burning, electric shocks, tingling) reported in 13.1% to 64.5% of patients, although heterogeneity in study design and tools used to assess this potential pain mechanism was high. Pain was frequently localized at the tumor site, although pain in other regions beyond the head and neck was also reported. Pain intensity was generally moderate, although varied across studies. OFP and headache in HNC patients were often neuropathic in nature and contributed significantly to disability and reduced quality of life. Most articles lacked mechanistic classifications of pain, although some suggested that central sensitization may be involved in some patients. Conclusions: Orofacial pain and headache are prevalent, under-characterized symptoms in HNC patients. There is an urgent need for standardized assessments using validated tools to improve phenotyping and inform targeted treatment strategies.

## 1. Introduction

Head and neck cancer (HNC) refers to a heterogeneous group of malignancies occurring in various regions, including the oral cavity, pharynx, larynx, salivary glands, and other related areas [1]. Worldwide, its prevalence has increased over the past decade, with nearly one million new cases diagnosed annually [2]. HNC is frequently associated with a long-lasting deterioration in quality of life [3]. Among the various signs and symptoms related to HNC and its treatment, pain is one of the most common, affecting 50–80% of patients [4,5].

Pain symptoms may result from tumor invasion, surgical trauma, radiation-induced tissue damage, or nerve injury, leading to a complex and often multifactorial pain experience [6,7]. Among the spectrum of pain symptoms in people with HNC, orofacial pain (OFP) and headache are particularly relevant due to their impact on quality of life. These symptoms can impair daily activities, such as eating, breathing, and speaking, and may even lead to reduced social interaction [8]. Headache is defined as “pain located in the head, above the orbitomeatal line and/or the nuchal ridge,” whereas OFP is defined as “a frequent form of pain perceived in the face and/or oral cavity,” making them anatomically distinct [9]. However, beyond these topographic differences, their underlying pathophysiological mechanisms and clinical implications also differ. Headaches may arise from primary origins (e.g., migraine, tension-type headache) or secondary causes, such as intracranial pathology or tumor invasion. In contrast, OFP encompasses a broader range of etiologies, including dental, musculoskeletal (e.g., temporomandibular disorders), neuropathic, or inflammatory sources. In the context of HNC, both headache and OFP can stem from direct tumor effects, treatment-induced damage, or neuroplastic changes. However, they may require different diagnostic approaches and management strategies. For this reason, although often reported together, distinguishing between them is essential to ensure accurate phenotyping and appropriate intervention in HNC populations [9].

In addition, the management plan of orofacial pain may be different depending on the clinical presentation and the source of symptoms, since different factors can be involved, such as dental diseases, myofascial pain syndromes, temporomandibular disorders, neuralgias, comorbidities, depression, and different pain mechanisms, such as nociceptive or neuropathic pain [10]. Nonetheless, despite the recognition of a poor understanding of orofacial pain in patients with head and neck cancer, some clinical features and mechanisms have been described. Nociceptive pain in HNC patients may arise from acute inflammation or tissue damage, typically described as sharp or throbbing. However, if left unresolved, particularly in cases involving surgical trauma or radiation-induced nerve injury, this may evolve into neuropathic pain, characterized by burning, tingling, or electric shocks. For instance, radiotherapy has been shown to cause neurotoxicity affecting cranial nerves or peripheral branches, thus contributing to the development of persistent neuropathic pain over time [11]. To frame this complexity, the IASP tripartite classification distinguishes nociceptive pain (due to tissue injury or inflammation), neuropathic pain (from somatosensory system damage), and nociplastic pain (from altered nociceptive processing without clear injury) [12]. In HNC patients, all three mechanisms may coexist due to tumor effects, surgery, or radiotherapy, yet this framework is rarely applied systematically in existing studies.

On another note, due to the different locations of tumors, cancer-related pain also presents considerable heterogeneity in its location, usually corresponding with the site of a tumor, but not exclusively, especially considering that sometimes widespread pain is present in people with cancer [13]. Moreover, controversy exists in differences between sexes, since while HNC usually affects more men than women, pain intensity may be more intense in women than in men [14].

Thus, despite their clinical relevance, the characteristics and mechanisms of orofacial pain and headache in HNC populations remain poorly defined and inconsistently assessed in the literature, leading to a lack of recognition of this condition, worse development of clinical strategies, and poorer prognostic outcomes [15]. Scoping reviews are particularly suitable for exploring broad or under-investigated topics in healthcare by mapping the existing literature and identifying key concepts, gaps, and types of evidence available [16]. To date, no comprehensive synthesis has systematically examined the clinical features of orofacial pain and headache in individuals diagnosed with HNC.

Despite the high prevalence and disabling nature of OFP and headache in individuals with head and neck cancer, the existing literature often overlooks the specific clinical features of these pain types. Most studies focus on general cancer-related pain without systematically differentiating clinical features in terms of location, descriptors, underlying mechanisms, or impact on quality of life. This lack of focused research leads to insufficient pain phenotyping and suboptimal clinical management. Furthermore, studies use heterogeneous assessment tools, and few apply standardized criteria to classify pain mechanisms (e.g., nociceptive vs. neuropathic), hindering data comparability and evidence-based intervention strategies. Thus, this review aims to identify and summarize the evidence describing the clinical features and pain mechanisms of orofacial pain and headache in people with HNC, performing an in-depth analysis of different characteristics, such as symptom descriptors. Such a synthesis will provide clinical tools to improve assessment and management, as well as act as the basis for future research aimed at improving headache-related outcomes in this population.

## 2. Materials and Methods

This scoping review was conducted to explore and synthesize the literature describing the characteristics of orofacial pain and headache in individuals diagnosed with HNC. The protocol for this scoping review was not registered with PRISMA ScR. It was registered in Open Science Framework (https://osf.io/z5yv2/, accessed on 1 July 2025), and it was developed following the framework proposed by Arksey and O’Malley [16] and further refined by Levac et al. [17]. To ensure the quality of reporting, the PRISMA extension for Scoping Reviews (PRISMA-ScR) checklist was used [18], although not registered with PRISMA ScR.

Following Arksey and O’Malley’s five-stage framework, this review included the following: (a) identifying the research question; (b) identifying relevant studies; (c) selecting the studies; (d) charting the data; and (e) collating, summarizing, and reporting the results.

### 2.1. Identifying the Research Question

The main research question was as follows: What are the clinical characteristics of orofacial pain and/or headache in individuals with head and neck cancer?

The sub-questions included the following:

- What are the typical frequency, intensity, quality, location, and duration of these pain symptoms?
- What mechanisms of pain are proposed (e.g., nociceptive, neuropathic, nociplastic)?
- What assessment tools are used to evaluate these types of pain in HNC populations?

This study was guided by the Participant–Concept–Context (PCC) framework. The population included adults diagnosed with any form of head and neck cancer; the concept was orofacial pain or headache, and the context included any clinical or research setting.

### 2.2. Identifying Relevant Studies

#### 2.2.1. Eligibility Criteria

We included original studies that reported the clinical features or mechanisms of orofacial pain or headache in individuals with head and neck cancer. We considered quantitative (e.g., observational, clinical trials) and qualitative designs. No time restrictions were applied. Inclusion was limited to studies published in English, Spanish, or French. Reviews, editorials, and conference abstracts were excluded unless they contained primary data not published elsewhere.

#### 2.2.2. Information Sources

A comprehensive search was conducted in the following databases until 15 June 2025: PubMed, Embase, Web of Science, and Scopus. Additionally, references of the included studies were manually screened to identify further relevant publications.

#### 2.2.3. Search Strategy

A sensitive and comprehensive strategy combining controlled vocabulary and free-text terms was developed for each database:

PubMed: The strategy used MeSH terms and free-text (e.g., “Head and Neck Neoplasms” [MeSH] AND (“Orofacial Pain” OR “Headache” OR “Neuropathic Pain”) AND (“Pain Measurement” OR “Pain Characteristics” OR “Pain Assessment”)).

Embase: The strategy used EMTREE terms and synonyms (e.g., ‘head and neck cancer’/exp AND (‘orofacial pain’/exp OR ‘headache’/exp OR ‘neuropathic pain’) AND (‘pain measurement’/exp OR ‘pain assessment’)).

Web of Science: The strategy used the Topic field (TS) for free-text search (e.g., TS = (“head and neck cancer”) AND TS = (“orofacial pain” OR “headache”) AND TS = (“pain characteristics” OR “pain assessment”)).

Scopus: TITLE-ABS-KEY (“head and neck cancer” OR “head and neck neoplasms”)

AND TITLE-ABS-KEY (“orofacial pain” OR “headache” OR “neuropathic pain”)

AND TITLE-ABS-KEY (“pain measurement” OR “pain assessment” OR “pain characteristics”).

### 2.3. Study Selection

After removing duplicates, the titles and abstracts were screened by two reviewers independently. Full-text articles were retrieved for potentially relevant records and assessed against the eligibility criteria. Discrepancies were resolved through discussion or adjudication by a third reviewer.

### 2.4. Data Charting

#### 2.4.1. Data Extraction

A standardized extraction form was developed to collect key information from the included studies. Two reviewers independently extracted the data. Any disagreement was resolved by consensus or consultation with a third reviewer.

#### 2.4.2. Extracted Variables

The following data were extracted:

- Study characteristics: author, year, and study design.
- Participant characteristics: cancer type, treatment status (pre-/post-therapy, during therapy), sample size, and sex.
- Pain characteristics: location, intensity, and frequency.
- Proposed pain mechanisms: nociceptive, neuropathic, nociplastic, or mixed.
- Affected activities and others.

### 2.5. Collating, Summarizing, and Reporting the Results

A descriptive and narrative synthesis of the included studies was conducted, organized by types of pain, cancer subgroups, or pain mechanisms where applicable. Key pain characteristics were summarized in tabular format. Since the primary aim of a scoping review is to map the available evidence rather than to appraise study quality, no formal risk of bias assessment was performed, consistent with the scoping review methodology [17,18].

## 3. Results

**Figure 1:**
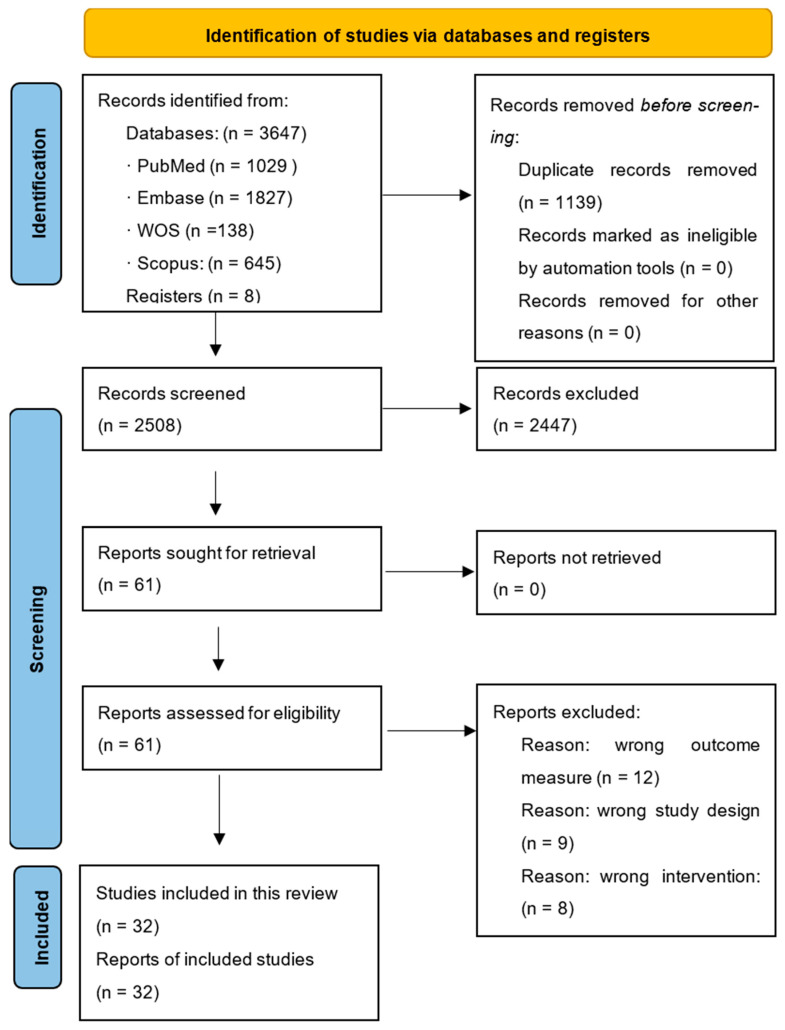
PRISMA Flow Diagram.

A total of 3647 records were identified, where 8 were identified through hand searching and the rest through electronisc database searches. After the removal of duplicates, 2508 unique records remained. The titles and abstracts were screened for eligibility, leading to the selection of 61 articles for full-text review. Of these, 32 studies met the inclusion criteria and were included in the final synthesis. The study selection process is illustrated in the PRISMA Flow Diagram (Figure 1).

A wide variety of findings were extracted. The characteristics of the assessed studies can be found in Table 1, while the orofacial and headache features can be found in Table 2.

- Type of study, sample size, and sex distribution.

The majority of the studies were observational in design, with 14 cross-sectional studies [22,25,27,32,34,35,36,37,38,39,45,48,49,51], 6 retrospective analyses [26,31,41,44,46,50], and 7 prospective observational studies [21,23,30,33,40,42,47]. Additionally, three randomized controlled trials (RCTs), one non-RCT [19,20,24,43], and one case series [29] were identified. Sample sizes varied considerably, ranging from 14 to 1002 participants. Most of the studies included a higher proportion of men than women, with up to 83% of the participants being male [24]. Only one study reported a nearly balanced sex distribution and an increased presence of females [35].

- Specific diagnosis and oncologic treatment status.

The most commonly reported diagnoses included oral squamous cell carcinoma and oropharyngeal carcinoma, although most of them were too general, with terms such as “oral cancer” or “HNC”. A few of the studies were more specific, such as one study investigating only those with nasopharyngeal carcinoma [46]. Moreover, other studies specified that the included patients were people with HNC and pain [19,23,26,43].

Regarding oncologic treatment, most of the studies were performed after the oncologic treatment was finished, with only six studies performed during the oncologic treatment [21,22,23,25,27,35], and nine studies were performed before the oncologic treatment [30,31,36,39,42,44,45,46,51].

- Outcome measures.

Pain assessment tools varied across the studies. The most frequently used instruments were VAS or NRS to quantify pain intensity. Concerning the type of pain, the most studied was neuropathic pain, with seven studies using questionnaires aimed to detect its presence, such as the S-LANSS [27,29,34,39,48] and the Douleur Neuropathique questionnaire [40,42].

Moreover, concerning quality of life, different questionnaires were used, such as the EORTC-QLQ 30 [30,37] and the UCSF-OCPQ [21,43,45]. Other questionnaires were included to assess craniomandibular function [37], pain catastrophism [35,37], and depression/anxiety [19,27].

- Pain characteristics: phenotype, location, intensity, and frequency.

Neuropathic pain features were reported in many of the studies, including symptoms such as burning, tingling, sharpness, electric shocks, and paresthesia. For example, in a prospective study [21], all patients had at least one neuropathic descriptor, with burning (21/22) and pins and needles (17/22) being the most frequent. Moreover, different proportions of patients with the presence of neuropathic pain were found, ranging from 13.1% [34] to 64.5% [29], with these differences possibly due to differences in study design, the use of different questionnaires, and the type of cancer or the timing of the assessment. For example, the studies using tools like S-LANSS or DN-4 tended to report higher rates of neuropathic pain than those relying solely on open-ended descriptors. However, not all the studies explicitly classified the pain phenotype.

The location of pain was most frequently reported in the orofacial region, particularly the tongue, buccal mucosa, floor of the mouth, and gingiva. Indeed, it was reported that 88% of patients presented with pain at the site of the tumor [34], although extraoral pain was also reported in sites such as the shoulders, chest, and extremities [21]. In that sense, it was reported that 36% of patients presented with widespread pain outside the head and neck region [33].

Pain intensity varied widely across the studies. Mild to moderate pain was most frequent, with VAS/NRS scores ranging from 3 to 6.4 in all the studies using these tools, except for one study, in which pain intensity was 1.51 [23]. When pain intensity was evaluated in terms of mild, moderate, or severe, we found considerable heterogeneity since, on the one hand, it was reported that up to 45% of patients had severe pain [29,36], but, on the other hand, the majority of the studies reported that severe pain was found in the lowest proportion of patients, with a higher prevalence of moderate intensity [27,30,34,37,38]. Interestingly, severe pain was more frequently reported in the studies assessing post-treatment populations [29,36], possibly reflecting chronic pain states associated with radiotherapy or surgery-related nerve damage. However, some variability may also reflect differences in the timing of assessment or the tools used.

Pain frequency was inconsistently reported, and a few of the studies recorded this feature. Nonetheless, continuous pain was reported in some of the studies with different percentages, such as 36% [29], 60% [26], and 93% [32]. Pain frequency was inconsistently reported across the studies, with some providing detailed percentages while others omitted this feature altogether, which limits comparability.

- Affected activities and other reported outcomes.

Pain impacted a wide range of functional activities, most notably chewing, speaking, opening the mouth, eating, swallowing, and social communication, with most patients among those with pain experiencing difficulty with chewing, jaw opening, and speaking [21]. Similarly, it was reported that 91.3% of participants reported interference with at least one of these activities [27].

On another note, other activities, such as sleep, were reported to be affected, with 55.4% of patients experiencing pain that disrupted sleep [36]. Other reported outcomes included emotional and psychological impacts, such as anxiety and depression [28,47,49], pain catastrophizing [35], and quality of life impairments [37,38]. For example, an association between radiation-induced chronic pain and sleep disturbances, anxiety, and depression was observed [38].

## 4. Discussion

This review aimed to synthesize the evidence regarding the main characteristics, features, and factors associated with orofacial pain and headache in people with HNC. To the best of our knowledge, no prior studies have performed such a comprehensive review concerning this topic. Our review shows considerable heterogeneity across studies, with few conclusions to be extracted. Nonetheless, we observed that some features, such as moderate pain intensity and the presence of neuropathic pain, may be common in this population.

Indeed, one of the most relevant findings is the importance of neuropathic pain (NP) characteristics in many HNC populations. Although sometimes, no validated questionnaires for the assessment of the presence/absence of neuropathic pain features were used, descriptors such as burning, tingling, and electric shocks were frequently reported, and up to 64.5% of patients met the criteria for probable NP in some samples. Moreover, it was also found that the intensity of pain was strongly correlated with the scores of the DN4 questionnaire, which could suggest that neuropathic pain may be involved in the intensity of headache {44]. This aligns with the neurotoxicity known to occur following radiotherapy and surgical interventions [28]. However, many studies failed to consistently classify pain mechanisms, limiting mechanistic insight and personalized treatment options.

Pain location was typically reported at or near the tumor site, which seems to be a logical consequence of radiotherapy, chemotherapy, and/or the surgical procedures [52]. Specifically, most reported regions were the tongue, floor of the mouth, and buccal mucosa [36,51], suggesting an important prevalence of pain after oncological treatment when cancer is present in these regions. Interestingly, some studies also reported extraoral and widespread pain distribution (e.g., upper extremities, chest), suggesting a potential central sensitization or widespread pain processing dysfunction, which remains underexplored in this population [53].

Another emerging observation is the evolution of pain profiles across treatment stages. While pre-treatment pain is often localized and potentially nociceptive in nature, post-treatment pain tends to show more neuropathic features, possibly linked to nerve injury caused by surgery or radiotherapy [54]. For instance, studies conducted after treatment reported higher pain intensities and greater use of descriptors like burning or electric shocks. This supports the hypothesis that pain trajectory in HNC is dynamic and shaped by therapeutic interventions, underscoring the importance of stage-specific assessment strategies [55].

Although limited by heterogeneity, some patterns emerged when considering treatment modality, tumor location, and disease timeline. For instance, studies assessing patients after radiotherapy more frequently reported neuropathic pain features, such as burning and electric sensations, likely reflecting treatment-induced neurotoxicity. In contrast, pre-treatment pain was often localized and described as nociceptive. Regarding cancer subsite, pain was more commonly reported in tumors of the oral cavity and oropharynx, particularly affecting the tongue and buccal mucosa, possibly due to their high innervation and exposure to mechanical stress. Additionally, pain intensity and functional impact were generally higher in post-treatment populations, suggesting a transition toward chronicity. These patterns underscore the need for stratified assessment and management strategies tailored to the stage of disease, treatment received, and anatomical site affected [56].

The impact on daily activities was substantial. Pain interfered with chewing, speaking, swallowing, and social communication. These functional limitations not only deteriorate quality of life but also correlate with psychological distress, including depression, anxiety, and catastrophizing. Indeed, it has been argued that the development and persistence of pain, due to the tumor itself, or to the treatments associated with its presence, might be accompanied by maladaptive cognitions, such as kinesiophobia or catastrophism [37,57]. This biopsychosocial impact underscores the need for multidimensional assessments and interventions. In that sense, psychological comorbidities such as anxiety, depression, and pain catastrophizing were common among patients with moderate-to-severe pain. Several studies showed significant correlations between emotional distress and pain severity [19,27,35,37], supporting the biopsychosocial model of cancer-related pain. These findings highlight the need for integrated care approaches that include psychological screening and support as part of pain management in HNC patients.

Although most studies reported a higher prevalence of head and neck cancer in men, few conducted sex-stratified analyses to examine potential differences in pain perception or pain mechanisms. Given previous findings suggesting that women may experience higher pain intensity and a greater prevalence of neuropathic features in other oncologic and non-oncologic populations [58], future studies should explore whether sex influences pain phenotypes, symptom trajectories, or treatment responses in patients with HNC. Such analyses could help identify sex-specific vulnerabilities and inform more personalized pain management strategies.

Regarding headache, few studies offered a precise diagnosis using ICHD-3 criteria [59]. Based on our results, we cannot identify if orofacial pain and headache present in people with HNC correspond with any specific type of headache. Indeed, the presence of pain descriptors was scarce, with, for example, only one study reporting the presence of unilateral pain in 65% of subjects [32]. Nonetheless, this may be related to the high heterogeneity in cancer subtypes.

Although not systematically assessed in most studies, the presence of widespread pain beyond the head and neck region, reported in up to 36% of patients [33], may suggest the involvement of central sensitization mechanisms. This pattern aligns with what has been described in other chronic pain conditions, where central nervous system hyperexcitability leads to amplified and spatially diffuse pain responses. The absence of validated tools for identifying central sensitization in the reviewed studies limits firm conclusions, but future research should consider incorporating instruments, such as the Central Sensitization Inventory [60], to better understand this dimension, or including psychophysical tests, such as conditioned pain modulation, to clinically assess the presence or absence of this physiological phenomenon [61]. Nonetheless, beyond peripheral and central mechanisms, the role of cranial autonomic structures, such as the sphenopalatine ganglion (SPG), should also be considered in the pathophysiology of orofacial pain and headache in HNC. The SPG has been implicated in several headache syndromes, including cluster headache and trigeminal autonomic cephalalgias, and may also contribute to referred facial pain via its connections with trigeminal and autonomic pathways. Recent evidence suggests that SPG-targeted interventions—such as blockades or neuromodulation—may modulate deep craniofacial nociceptive signaling and offer therapeutic potential in complex pain presentations, including those found in head and neck cancer survivors [62]. Incorporating SPG-related mechanisms into future studies may enhance our understanding of pain phenotypes and open new avenues for individualized pain management.

### 4.1. Limitations

This review is not without limitations. At first, the heterogeneity in different characteristics, including study design, cancer subtypes, pain mechanisms, pain features, such as intensity, and outcome measures, made it difficult to arrive at firm conclusions. For example, although some studies used standardized pain assessment tools, such as the S-LANSS questionnaire, many studies did not, which could lead to potential misclassifications. Secondly, most data came from cross-sectional or retrospective studies, limiting causal inferences. Thirdly, few studies stratified data by sex, age, or psychosocial variables, despite their known influence on pain experiences. Furthermore, potential publication bias should be considered, as most of the included studies originated from similar geographic regions or specialized oncology centers, which may not reflect broader or underserved populations. This may limit the generalizability of the findings, particularly in settings with different healthcare infrastructures or cultural pain expressions. Moreover, although risk of bias assessments are not mandatory in scoping reviews, the predominance of cross-sectional and retrospective designs among the included studies introduces the possibility of selection, recall, and reporting biases. These methodological limitations may affect the accuracy of pain prevalence estimates and the consistency of reported pain characteristics. For example, self-reported measures may be influenced by memory distortions or emotional state, and studies conducted in specialized settings may select for more severe or treatment-resistant cases. Therefore, the findings should be interpreted with caution and considered exploratory rather than definitive. In addition, we restricted inclusion to articles published in English, French, or Spanish. While this decision was based on the authors’ language proficiency to ensure accurate interpretation of the results, it may have introduced language bias. This is particularly relevant given that pain perception, expression, and documentation may vary across cultures and healthcare systems, potentially leading to underrepresentation of data from non-Western countries, especially in Asia. Finally, the description of pain characteristics was limited, with few studies recording features such as frequency.

### 4.2. Future Directions

Future research should employ prospective designs to track pain evolution from the very beginning to diagnosis and through survivorship. Moreover, the use of standardized methods for reporting pain features could help to achieve more comprehensive assessments and, thus, data comparison. In addition, it could lead to the identification of headache subtypes in this population based on the ICHD-3 [58], which could help in headache management.

## 5. Conclusions

Orofacial pain and headache are highly prevalent and clinically impactful in people with head and neck cancer. Neuropathic features are frequent, particularly following radiotherapy, and significantly impair functional activities and quality of life. However, pain characterization remains inconsistent, and our mechanistic understanding is limited. A standardized, multidimensional approach to pain assessment in this population is urgently needed.

## Figures and Tables

**Table 1:** Study characteristics.

Study	Type of Study	Specific HNC Diagnosis	Sample Size	Sex (F/M)	Oncologic Treatment Status
Yang et al., 2017 [19]	Non-RCT	OSCC	124	21/103	During treatment
Haumann et al., 2018 [20]	RCT	HNC with nociceptive pain	82	32/50	During treatment
Ouadghiri et al., 2024 [21]	Prospective observational study	Oral or oropharyngeal cancer	17	6/11	Post-treatment (4 years)
Sato et al., 2010 [22]	Cross-sectional study	OSCC	113	31/82	Pre-treatment
Ye et al., 2023 [23]	Prospective study	Oral cavity, oropharynx, or larynx	25	6/19	During treatment
Shinde et al., 2023 [24]	RCT	Oral cancer survivor with TMJD	100	26/74	Post-treatment
Schaller et al., 2021 [25]	Cross-sectional study	HNC with oral mucositis	63	24/39	During treatment
Saghafi et al., 2021 [26]	Retrospective	HNC in the tonsil and the base of the tongue	217	57/160	Post-treatment
Ortiz-Comino et al., 2019 [27]	Cross-sectional study	HNC with oral cavity, pharyngeal, laryngeal, salivary gland, or nasal cavity cancer	30	10/20	During treatment
Arantes et al., 2018 [28]	Case series	HNC and OFP	22	4/18	Post-treatment
Jiang et al., 2019 [29]	RCT	HNC with radiotherapy-related neuropathic pain	128	51/77	Post-treatment
Epstein et al., 2009 [30]	Prospective observational study	HNC with pain	124	29/95	During treatment
Chua et al., 1999 [31]	Retrospective study	HNC with pain	40	-	Post-treatment
Sarumpaet et al., 2023 [32]	Cross-sectional study	HNC detected through histopathology	127	46/81	During treatment
Aghajanzadeh et al., 2023 [33]	Prospective observational study	HNC	194	38/146	Pre-treatment
Khawaja et al., 2023 [34]	Cross-sectional study	HNC-related pain according to the International Headache Society	100	32/68	-
Rojo et al., 2022 [35]	Cross-sectional study	HNC survivors	505	113/392	Post-treatment
Siegel et al., 2022 [36]	Cross-sectional study	HNC with tumor in the sellar region	112	59/53	During treatment
Vilarim et al., 2022 [37]	Cross-sectional study	HNC	74	15/59	Pre-treatment
Arranz-Martin et al., 2024 [38]	Cross-sectional study	HNC	78	26/52	Pre-treatment, during treatment, and post-treatment
Zuo et al., 2024 [39]	Cross-sectional study	HNC with radiotherapy-related neuropathic pain	1002	288/714	Post-treatment
Lou et al., 2021 [40]	Prospective longitudinal study	HNC	77	24/53	Pre- and post-treatment
Khawaja et al., 2021 [41]	Retrospective study	Oral cancer (90.2% OSCC)	1067	385/682	Pre-treatment
Kouri et al., 2021 [42]	Prospective observational study	HNC	26	6/20	Post-treatment
Salwey et al., 2020 [43]	Prospective observational study	OSCC	60	17/43	Pre-treatment
Kallurcar et al., 2019 [44]	Retrospective study	HNC	53	14/39	Post-treatment
Buchakjian et al., 2017 [45]	Cross-sectional study	HNC	27	7/20	Pre-treatment
Van Abel et al., 2014 [46]	Retrospective study	HNC	38	14/24	Pre-treatment
Lee and Ho, 2012 [47]	Retrospective study	Nasopharyngeal carcinoma	14	4/10	Pre-treatment
Lam and Schmidt, 2011 [48]	Cross-sectional study	Oral cancer	44	19/25	Pre-treatment
Potter et al., 2003 [49]	Cross-sectional study	HNC	25	11/14	Post-treatment
Vecht et al., 1993 [50]	Cross-sectional study	HNC	25	11/14	Post-treatment

HNC: head and neck cancer; OFP: orofacial pain; OSSC: oral squamous cell carcinoma; RCT: randomized-controlled trial; TMJD: temporomandibular joint dysfunction.

**Table 2:** Orofacial and headache features.

Study	Outcome Measures	Pain Characteristics/Phenotype	Location	Intensity	Frequency	Affected Activities in Relation to Pain	Others
Yang et al., 2017 [19]	UCSF-OCPQ (0–100 mm)	Spontaneous sharpness: 15.6 (12.85) Functional sharpness: 20.87 (13.03) Spontaneous aching: 33.33 (13.54) Sensitivity to touch: 44.87 (21.13) Functional restriction: 46.2 (16.2)	-	Spontaneous intensity: 29.53 (14.80) Functional intensity: 32.73 (16.34)	-	-	-
Haumann et al., 2018 [20]	NRS (0–10); HADS	-	-	5.4 (1.9)	-	-	Anxiety: 52% Depression: 21%
Ouadghiri et al., 2024 [21]	VAS (0–100)	Two with neuropathic and spontaneous pain Only one with paresthesia	-	With pain: 2/17 Mean intensity: 4.5	-	-	-
Sato et al., 2010 [22]	Location of pain	Spontaneous pain: 37% Function-related pain: 68%	Tongue: 34%; lower gingiva: 11%; buccal mucosa: 8%; floor of the mouth: 2%; upper gingiva: 4%	-	-	-	-
Ye et al., 2023 [23]	UCSF-OCPQ (0–10)	Pain in 88% All at least one NP descriptor, 54% two Burning: 21/22 Pins and needles: 17/22 Tingling: 2/22 Numbness, itching pinching, and shooting: 1/22	With pain at the site of tumor: 88% With pain in other orofacial region: 36%	5.4	-	Chewing, (15), mouth/jaw opening (12), talking (13), eating (12), drinking (9)	Advanced clinical stage of tumor is related to pain severity
Shinde et al., 2023 [24]	VAS (0–100)	-	-	4.34	-	-	-
Schaller et al., 2021 [25]	NRS (0–10)	-	-	3.0	-	-	-
Saghafi et al., 2021 [26]	-	Symptom from the TMJ + pain upon palpation: 2/217 Symptom from the muscles + pain upon palpation: 12/217 Pain upon chewing: 4/217 Pain upon mouth opening: 2/217	TMJ (23/217)	-	-	-	-
Ortiz-Comino et al., 2019 [27]	VAS (0–10)	-	Cervical	Cervical: 3.5 (3.3) TMJ: 2.4 (3.4)	-	-	-
Arantes et al., 2018 [28]	VAS (0–10); LANSS	Nociceptive: 45.4% Neuropathic: 18.1% Mixed: 36.3% Pain sensations: stinging (36.3%), throbbing (27.2%), pressure (18.1%), burning (18.1%), needle prick (9%), tingling (4.5%), painful cold sensation (4.5%)	-	Severe pain: 45.5% Moderate pain: 22.7% Mild pain: 31.8%	Continuous pain in 36.3%	-	-
Jiang et al., 2019 [29]	NRS; BPI; POMS	-	-	6.4	-	-	Pain interference and psychological distress associated with pain intensity
Epstein et al., 2009 [30]	McGill Pain Questionnaire Pain intensity (0–5)	Aching: 20.2%; boring: 5.6%; burning: 26.6%; drilling: 2.4%; flashing: 4.0%; freezing: 0.8%; hot: 9.3%; lancinating: 0.8%; pricking: 1.6%; beating: 3.2%; dull: 21.8%; hurting: 12.9%; pressing: 10.5%; sharp: 15.3%; rasping: 8.1%; pulsing: 7.3%	Head: 98/124 Arm: 3/124 Abdomen: 1 Back: 3 Missing/unknown: 19	1.51 (1.01)	-	-	Other affective and evaluative descriptors were included, such as tiring (25%) or exhausting (8.9%)
Chua et al., 1999 [31]	BPI; NRS	Somatic: 13 Neuropathic: 3 Somatic + neuropathic: 15 Myofascial: 6 Others: 3	Frontal region: 17 Maxillofacial region: 50 Anterior neck: 13 Posterior neck: 10 Occipito-parietal region: 9	5.7 (2.5)	Continuous: 60%	-	Trigeminal nerve was the most frequently affected nerve (16)
Sarumpaet et al., 2023 [32]	VAS; BPI; HADS; LANSS	Neuropathic pain: 65.4%	-	Moderate pain: 53.5%; severe pain: 26%; mild pain: 20.5%	-	Overall, 91.3% experienced interference while eating, speaking, chewing and/or opening the mouth	Overall, 64.6% experienced depression
Aghajanzadeh et al., 2023 [33]	Likert Scale for pain intensity; EORTC-QLQ 30	-	A total of 97 presented with facial pain	Mild pain: 55%; moderate: 33%; severe: 12%	-	-	Pain intensity was correlated with less mouth opening
Khawaja et al., 2023 [34]	NRS	Sharp: 29; pressing: 1; electric: 22; burning: 57; throbbing: 24; stinging: 19; dull ache: 86; stabbing: 22 Myofascial pain: 81; jaw bone pain disorder: 53; burning pain disorder: 40; neuropathic: 20; unknown: 16	Unilateral: 65%	5.9 (2.5)	Constant: 93%	-	-
Rojo et al., 2022 [35]	NRS; S-LANSS	Neuropathic pain: 13.7%	Head and oral cavity: 46.2% Neck and throat: 41.5% Shoulders: 24.6% Upper extremities: 26.2% Anterior chest: 1.5% Posterior chest: 10.8% Abdomen: 1.5% Lower back and pelvis: 24.6% Lower extremities: 24.6%	Mild: 31% Moderate: 52% Severe: 17%	-	-	Severe pain was more frequent in those with neuropathic pain
Siegel et al., 2022 [36]	PCS; EHI	Migraine without aura: 47%; migraine with aura: 27%; chronic daily headache: 29%; tension-type headache: 27%; cluster headache: 11%; medication induced headache: 4%; not classifiable: 18%	Frontal: 53%; holocranial: 14%; occipital: 6%; right side: 4%; various locations: 22%	-	.	-	Headache occurrence was associated with catastrophism
Vilarim et al., 2022 [37]	Verbal scale of pain; Standardized Evaluation of orofacial pain	Orofacial pain: 91.9% Jumping: 51.4%; burning: 45.9%; throbbing: 40.5% Toothache-like: 10.8%.	Mouth: 28.4%; tongue: 40.5%; throat: 16.2%; ear: 10.8%; palate: 8.1%; face: 8.1%; tooth: 4.1%; gingiva: 4.1%; neck: 2.7%	Mild: 16.2%; moderate: 29.7%; severe: 45.9%	Morning: 1.4%; afternoon: 6.8%; night: 6.8%; variable: 77%	Triggering factors: chewing: 16.2%; speaking: 1.4%; swallowing: 10.8%	Overall, 55.4% reported that pain woke up them during the night
Arranz-Martin et al., 2024 [38]	EORTC QLQ; PCS; NRS; TSK-TMD; CF-PDI-11	-	-	Mild: 21.8%; moderate: 15,4%; severe: 3.8%	-	-	Kinesiofhobia was correlated with low ranges of mouth opening
Zuo et al., 2024 [39]	NRS; SAS; SDS; WHOQOL-BREF	Radiotherapy-induced chronic pain: 67.1%	-	Mild: 41.6%; moderate: 18.9%; severe: 11.3%	-	Insomnia: 22.5%	Radiation-induced chronic pain increased risk of anxiety, depression, sleep, and QOL
Lou et al., 2021 [40]	VHNSS; SF-MPQ; 2.4.4|Head and Neck Lymphedema and Fibrosis Symptom Inventory	Chronic pain joint (pain/arthritis, low back pain, neck pain, and facial/jaw pain): 40% Nociceptive pain from mouth and throat sores (largely due to mucositis) was most prevalent at end of treatment and resolved by 3–6 months: 54.5%	Widespread pain outside of the head and neck region: 36%; at 12 months: 40%	Moderate to severe (4–10): 31%; at 12 months: 40%	-	Mucosal and dental sensitivity on dental hygiene and eating: 39.7% at 3 months and 23% at 12 months	Patients with pain at 3–6 months post-treatment were likely to have transitioned to a chronic pain state
Khawaja et al., 2021 [41]	NRS	Chronicity of pain Pain <3 months: 228 (33.7%) Pain ≥3 months: 448 (66.3%)	Tongue: 55.3% Buccal mucosa: 22% Maxillary or mandibular alveolar ridges: 16.1%	Pain: 67.5% Moderate to severe (4–10): 42%	Intermittent: 31%	Mouth Opening: 10% Restricted tongue mobility with interference with mastication, speech, and swallowing 12.5%	-
Kouri et al., 2021 [42]	NRS; DN-4; EORTC	Neuropathic pain, descriptors: “burning” (34.62%), “electric shocks” (30.77%), and “pins and needles” (30.77%)	-	Overall, 65% developed moderate or severe pain (NRS ≥ 5)	-	-	Strong correlation between DN4 questionnaire scores and the intensity of oral mucositis
Salwey et al., 2020 [43]	NRS; DN-4; NPSI	NP+: 24 (40%) NP−: 36 (60%)	-	Mild: 20%; moderate to severe: 53%	Pain/24 h: Permanent: 8 (33%); 8–12 h: 4 (17%); 4–7 h: 3 (12.5%); 1–3 h: 6 (25%); <1 h: 3 (12.5%). Episodes/24 h: 20: 1 (4%); 1–20: 6 (25%); 6–10: 7 (29%); 1–5: 6 (25%). Absent: 4 (17%).	-	-
Kallurcar et al., 2019 [44]	NRS	Chronic NP with descriptors of neuropathic pain, such as stinging or burning pain: 32 (60%)	Oral cavity: 24.5% Pharynx: 26.4% Eyes: 1.9% Nasal cavity/paranasal sinuses: 1.9% Odynophagia: 17% Headache: 5.7% Face: 9.4% Ear: 15.1% Neck: 15.1%	-	-	-	Significant association between chronic pain and number of pain sites
Buchakjian et al., 2017 [45]	S-LANSS; HADS; WPI/SS; BPI	NP+: 38%	Jaw: 62% Ears: 52% Tongue: 29% Other: 76%	With pain: 21/27 Mean intensity: 4	-	-	Anxiety: 52% Depression: 30% + Fibromyalgia: 0%
Van Abel et al., 2014 [46]	-	Sharp: 33 (86.8%) Dull: 4 (10.5%) Burning: 1 (2.6%)	-	Mild: 2.6%; moderate: 52.6%; severe: 44.8%	Constant: 6 (15.8%) Intermittent: 32 (84.2%)	Dysphagia: 21%	-
Lee and Ho, 2012 [47]	Location and pain characteristics	Headache: 100% Dull: 3 Pulsating: 3 Explosive: 2 Pressing: 2 Tightening: 1 Debilitating: 1 Pressure-like: 1 Nonspecific: 1	Temporal: 6 Parietal: 3 Frontal: 1 Diffuse: 4	-	-	-	Overall, 71% had improved headache during or after treatment
Lam and Schmidt, 2011 [48]	UCSF-OCPQ	-	-	Moderate to severe (>30 mm): 84%	-	-	-
Potter et al., 2003 [49]	LANSS	NP+: 56%	-	-	-	Swallowing warm or cold drinks: 16%	-
Vecht et al., 1993 [50]	NRS	Nociceptive pain: 9 (36%) Nociceptive nerve pain: 8 (32%) Referred: 2 (8%) Neuropathic pain: 6 (24%)	-	All patients > 50; 80% patients > 70	Constant: 19 (76%) Intermittent: 6 (24%)	-	-

BPI: Brief Pain Inventory; DN-4: Douleur Neuropathique 4; EHI: Essen Headache Inventory; EORTC QLQ30; HADS: Hospital Anxiety and Depression Scale; LANSS: Leeds Assessment of Neuropathic Sygns and Symnptoms; NP: neuropathic pain; NRS: Numerical Rating Scale; PCS: Pain Catastrophism Scale; POMS: Profile of Mood States; UCSF-OCPQ: The University of California San Francisco Oral Cancer Pain Questionnaire; VAS: Visual Analogue Scale; CF-PDI-11: Short Version of the Craniofacial Pain and Disability Inventory; SAS: Self-rating Anxiety Scale; SDS: Self-rating Depression Scale; WHOQOL-BREF: World Health Organization Quality of Life Assessment-Brief; VHNSS: Vanderbilt Head and Neck Symptom Survey; SF-MPQ: Short-Form McGill Pain Questionnaire; NPSI: Neuropathic Pain Symptom Inventory.

## Data Availability

Not applicable.
